# Supplementary material for: MetaRibo-Seq measures translation in microbiomes
Source: Nat Commun. 2020 Jun 29;11:3268. doi: 10.1038/s41467-020-17081-z (PMC7324362; doi:10.1038/s41467-020-17081-z)
Supplement: Supplementary file 10 — Supplementary Data 7 [file 41467_2020_17081_MOESM10_ESM.zip › File2/Confidence_High_Taxonomy/62060_out.krona.html]

Javascript must be enabled to view this page.

members
magnitude
magnitudeUnassigned
count
unassigned
taxon
rank

62060\_out

9

2
superkingdom
9

phylum
976
9

9
200643
class

9
171549
order

9
171552
family

9
838
genus

species
165179

SRS012969\_contig\_number\_3083SRS015794\_contig\_number\_10174SRS023526\_contig\_number\_contig-100\_726.226246SRS043667\_contig\_number\_2498SRS049959\_contig\_number\_706SRS049959\_contig\_number\_contig-100\_27867.158429SRS077849\_contig\_number\_contig-100\_89.185858
7


SRS012849\_contig\_number\_contig-100\_4276.193798SRS023715\_contig\_number\_9095
2
2292054
species
